# Metabolic syndrome increases risk for perioperative outcomes following posterior lumbar interbody fusion

**DOI:** 10.1097/MD.0000000000021786

**Published:** 2020-09-18

**Authors:** Xiaoqi He, Qiaoman Fei, Tianwei Sun

**Affiliations:** a Department of Orthopedics, Xianyang Central Hospital, Xianyang, Shaanxi; b Graduate School of Tianjin Medical University, Tianjin; c Department of Spinal Surgery, Tianjin Union Medical Center, Tianjin, People's Republic of China.

**Keywords:** complications, length of stay, lumbar spine, metabolic syndrome, spinal fusion

## Abstract

The present study is a retrospective cohort study. Metabolic syndrome (MetS) is a clustering of clinical findings that has been shown to increase the risk of the surgical outcomes. Our study aimed to evaluate whether MetS was a risk factor for increased perioperative outcomes in patients undergoing posterior lumbar interbody fusion (PLIF).

We retrospectively analyzed patients over 18 years following elective posterior lumbar spine fusion from January 2014 to December 2018. Emergency procedures, infections, tumor, fracture, and revision surgeries were excluded. Patients were divided into 2 groups with and without MetS. The MetS was defined by having 3 of the following 4 criteria: obesity (body mass index ≥30 kg/m^2^), dyslipidemia, hypertension, and diabetes. The follow-up period lasted up to 30 days after surgery. The outcomes of demographics, comorbidities, perioperative complications, and length of stay were compared between the 2 groups. Multivariate logistic regression analysis was used to identify perioperative outcomes that were independently associated with MetS.

The overall prevalence of MetS was 12.5% (360/2880). Patients with MetS was a significantly higher risk factor for perioperative complications, and longer length of stay cmpared with patients without MetS (*P* < .05). The MetS group had a higher rate of cardiac complications (*P* = .019), pulmonary complication (*P* = .035), pneumonia (*P* = .026), cerebrovascular event (*P* = .023), urinary tract infection (*P* = .018), postoperative ICU admission (*P* = .02), and deep vein thrombosis (*P* = .029) than non-MetS group. The patients with MetS had longer hospital stays than the patients without MetS (22.16 vs 19.99 days, *P* < .001). Logistic regression analysis revealed that patients with MetS were more likely to experience perioperative complications (odds ratio [OR] 1.31; 95% confidence interval [CI]: 1.06–2.07; *P* < .001), and extend the length of stay (OR: 1.69; 95% CI: 1.25–2028; *P* = .001).

The MetS is a significant risk factor for increased perioperative complications, and extend length of stay after PLIF. Strategies to minimize the adverse effect of MetS should be considered for these patients.

## 1 Introduction

The metabolic syndrome (MetS) encompasses a group of medical conditions that increase the risk of cardiovascular disease, and all-cause mortality during an individual's life.^[1]^ The MetS has been indicated as a risk factor for developing these perioperative complications,^[2,3]^ such as increased the difficulty of exposure, operative time, and wound complications.^[4,5]^

There are some differences in the definition of MetS as to its components and their individual contribution in establishing the diagnosis.^[6]^ At present, it is widely believed that obesity, hypertension, elevated fasting glycemia, and dyslipidemia are the main components of the MetS.^[7]^ Thus, the pathogenesis of MetS is complex and multifactorial, which includes sedentary lifestyle, obesity, diet, and genetic predisposition.

In orthopedic surgery, some studies have found MetS to be an independent predictor of adverse outcomes postoperatively.^[6]^ Utilizing the National Quality Surgical Improvement Project (NSQIP) database, Chung et al^[8]^ reported that patients with MetS who underwent lumbar spinal fusion was a significant increase in the incidence of pulmonary complications (*P* = .048), sepsis (*P* = .039), and acute postoperative renal failure (*P* = .001), and an increase in hospital length of stay (4.38 vs 3.81 days), but found no association with 30-day readmissions and reoperations.

Passias et al^[9]^ found that the patients with MetS had higher average total costs of surgery compared non-MetS patients ($60,579.30 vs. $52,053.23, *P* < .05) following spine fusion surgery. This study further identified that MetS increased 50% higher costs per quality adjusted life years at 1 year, and 75% higher cost per quality-adjusted life years.

According to relevant reports, the prevalence of the MetS is known to increase with age: although only 6.7% of patients between the ages of 20 and 29 years were affected; 43.5% of patients aged 60 to 69 years and 42% of patients 70 years and older are estimated to have MetS.^[10]^

The number and proportion of posterior lumbar interbody fusion (PLIF) patients with MetS is likely to continue to rise given the worsening worldwide obesity epidemic and an aging population. So, it is important to assess the effects this syndrome could have on perioperative outcomes. However, there are few studies on perioperative outcomes in patient with MetS after PLIF. We aimed to identify whether MetS was an independent risk factor for increased major perioperative complications and extended hospital length of stay following elective posterior lumbar fusion surgery.

## 2 Methods

This study was a retrospective cohort study of a database containing spine patients presenting to a single academic institution from January 2014 to December 2018. All patients who underwent primary PLIF gave informed consent to participate in this study, which was reviewed and approved by the local institutional ethics committee. During the study period, only the data from the most recent surgical procedure were included to avoid potential bias. The entry about the performance of PLIF was identified using the *International Classification of Diseases, Ninth Revision, Clinical Modification* (*ICD-9-CM*). The study population was divided into 2 groups (MetS and non-MetS).

All patients met the following inclusion criteria: the subjects in the study were adults (age >18); patients with legally competent to consent, and receiving general anesthesia; surgical indications mainly included degenerative diseases of the spine such as degenerative disc disease, lumbar spinal stenosis, degenerative scoliosis. The exclusion criteria were as follows: the patients with incomplete information, severe organs insufficiency, pregnancy; the patients with a nonclean wound, an open wound on their body, preoperative sepsis, preoperative pneumonia, previous surgery within 30 days, cardiopulmonary resuscitation before surgery, and patients preoperatively admitted to the intensive care unit (ICU); the cases with no-spine surgery, immunodeficiency; the surgical indications involved emergency procedures, infections, tumor, fracture fixation, and revision surgeries.

### 2.1 Primary outcome

We collected demographic and comorbidity data, height, weight, preoperative laboratory results, complications, and length of stay. The follow-up period lasted up to 30 days after surgery. Operative data included surgical indication (such as degenerative disc disease, lumbar spinal stenosis, degenerative scoliosis), ASA grade, number of levels fused (<3 levels, >3 levels), operative time, transfusion, and blood loss. The comorbidities included diabetes, obesity, dyslipidemia, hypertension, chronic pulmonary disease, coronary artery disease, neurologic, renal and peripheral vascular disease. The perioperative complications included deep vein thrombosis (DVT), pulmonary embolism (PE), reoperation, requiring intensive care unit transfer (ICU), acute renal failure, urinary tract infection, pneumonia, surgical site infection (SSI), and myocardial infarction (MI). Demographic variables were analyzed, including age (18–49 years, 50–69 years, 70–79 years, ≥80 years), sex, body mass index (BMI), smoking history, blood pressure, fasting plasma glucose, triglycerides, and high-density lipoprotein cholesterol.

### 2.2 Laboratory evaluation

The BMI was computed as weight/height squared. Systolic and diastolic blood pressure levels were then read 3 times at 1-minute interval, and the mean of the second and third readings was used in the analysis. Plasma lipid and glucose levels were measured by routine assays. We described the variation trend of the incidence of MetS as well as the components of MetS through the line chart, bar chart, and pie chart.

### 2.3 The Definition of MetS

The US National Cholesterol Education Program Adult Treatment Panel III (NCEP-ATP III)^[11]^ defined the MetS as the presence of 3 of the following 5 conditions: a waist circumference of higher than 88 cm for females and higher than 102 cm for males; an arterial blood pressure of 130/85 mmHg or higher or the current use of antihypertensive medication; a plasma triglyceride level of 150 mg/dL or higher; a serum high-density lipoprotein (HDL) cholesterol level of <50 mg/dL for females and <40 mg/dl for males; and a fasting serum glucose level of ≥110 mg/dL or a clinical diagnosis of diabetes with dietary, oral, or insulin treatment.

However, we did not obtain the data on abdominal circumference, and used the BMI instead of waist circumference. Accordingly, the MetS was defined as the presence of 3 of the following 4 criteria: obesity (BMI ≥30 kg/m^2^), dyslipidemia, hypertension, and diabetes.

### 2.4 Statistical analysis

The continuous variables were presented as mean values ± standard deviation; the categorical variables were described using frequency distributions, and they were reported as percentages. The student *t* test was used for comparisons between normally distributed continuous variables. A *χ*^2^ test was used to compare categorical demographics between the 2 groups. Multivariate logistic regression analysis was performed to identify if MetS was a risk factor for perioperative complications, and prolonged length of stay. When the average length of stay was greater than the 75th percentile, they were defined as prolonged length of stay. A *P* value of .05 was considered significant. All statistical analyses were performed with SPSS version 22.0 software (SPSS Inc, Chicago, IL).

## 3 Results

**Figure 1:**
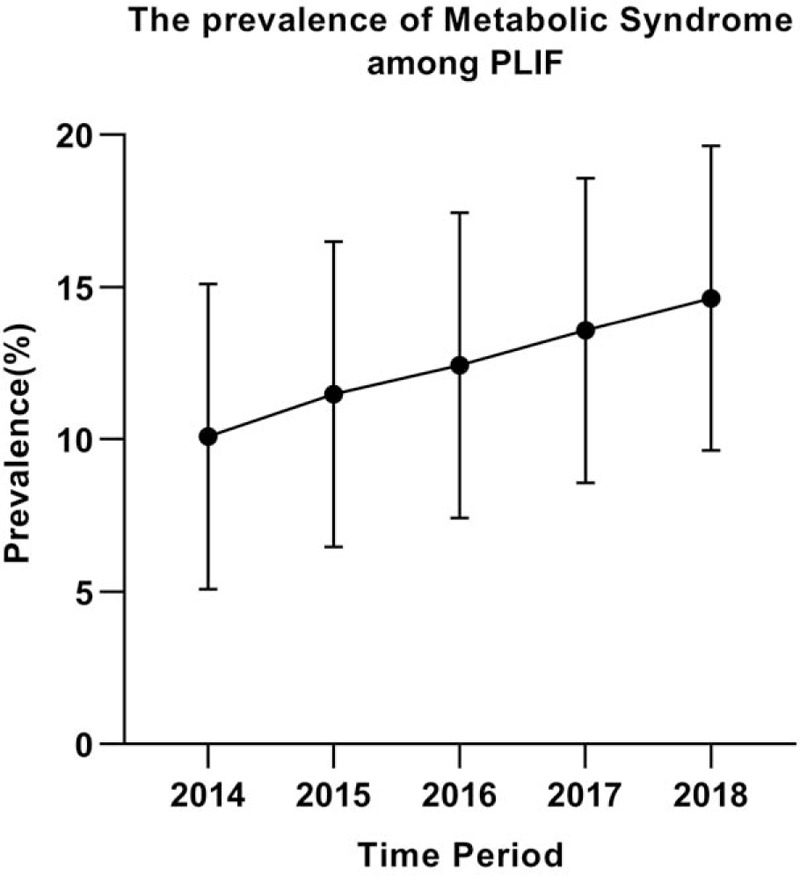
The prevalence of metabolic syndrome (MetS) between 2014 and 2018 among patients for primary posterior lumbar spine fusion. The prevalence of MetS for posterior lumbar interbody fusion (PLIF) admissions increased over time and reached 14.63% in the most recent period, respectively.

A total of 2880 patients were identified between the 2014 and 2018 follow-ups. The average prevalence of MetS over the entire study period was 12.5% of patients, with a peak of 14.63% in 2018 (Fig. 1). The mean age of all patients was 59.4 ± 9.8 years. There were 1511 females and 1369 males. Patients with MetS were older (*P* < .001), and a greater smoking population (*P* = .034), compared with the non-MetS group. Patients without MetS had an average BMI of 26.89 kg/m^2^, whereas the average BMI for patients with MetS was 30.88 kg/m^2^. There were no significant difference in sex, surgical time, blood loss, transfusion, levels fused, and spine pathology between MetS and non-MetS group (Table 1).

**Table 1:**
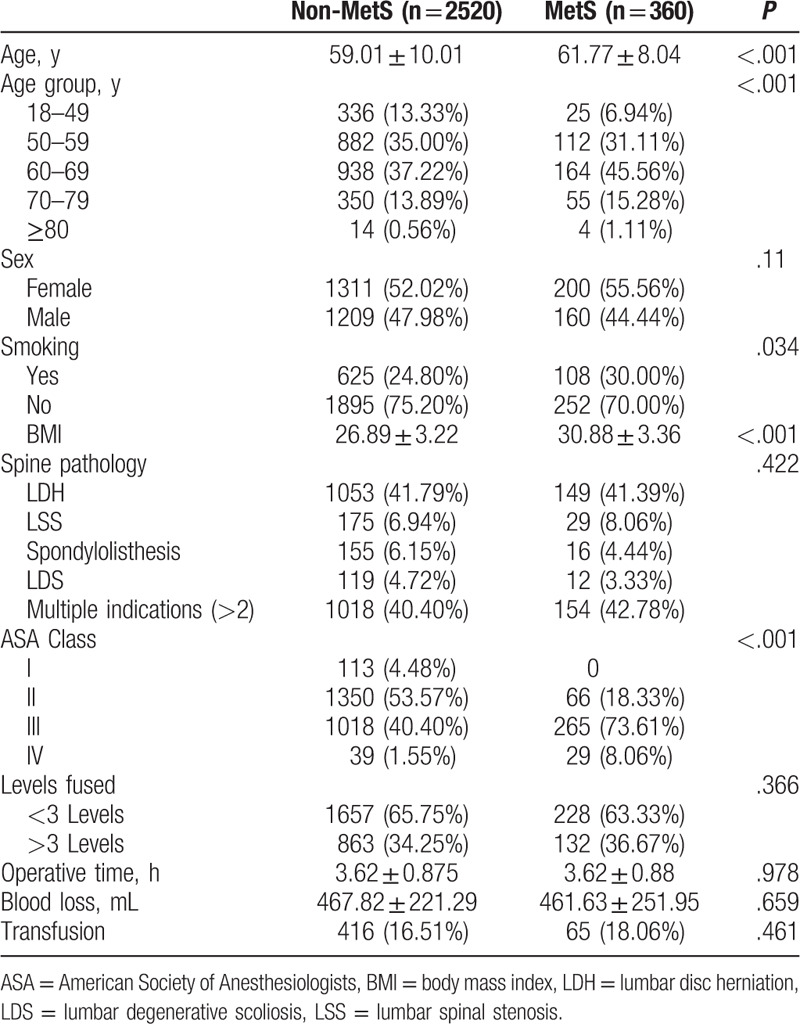
Demographic and clinical characteristics of patients with and without metabolic syndrome in the study population.

**Figure 2:**
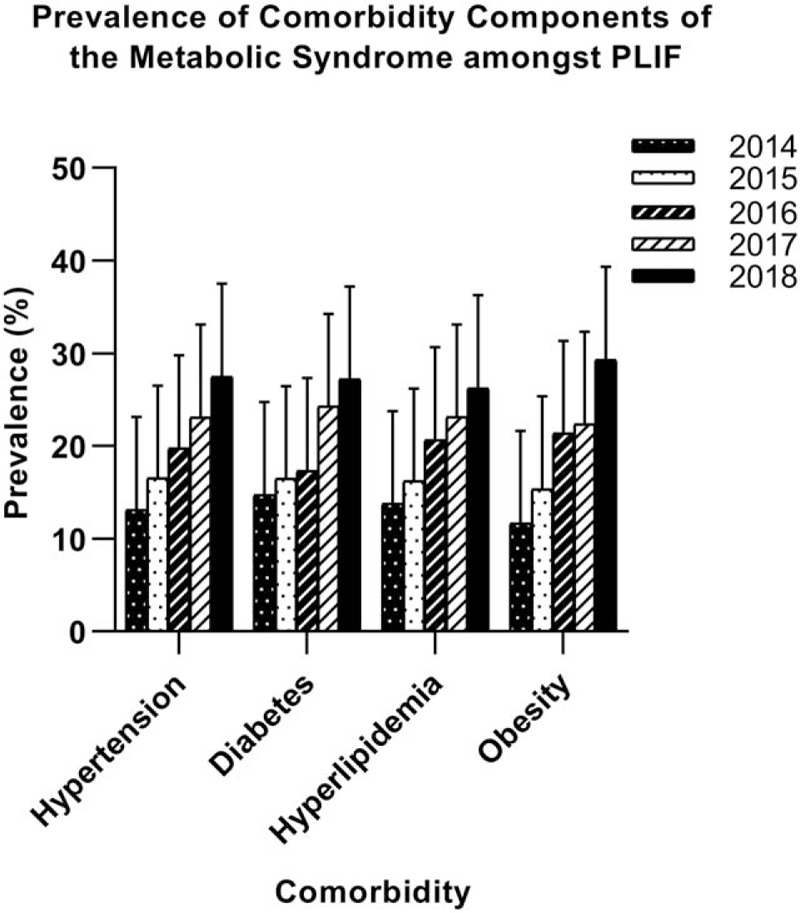
The prevalence of metabolic syndrome component comorbidities for patients undergoing posterior lumbar spine fusion over time. An increase in the comorbidity components of the prevalence of metabolic syndrome was detected between the time periods 2014 and 2018.

**Figure 3:**
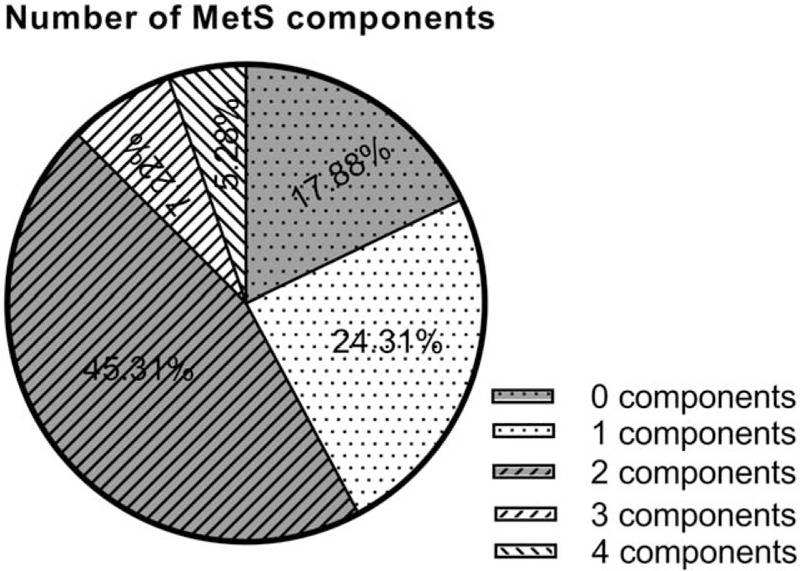
The graph showing patient distribution according to the positive numbers of components of the metabolic syndrome. It is shown that the highest of the 2 components was 45.31% and the lowest proportion of the four components was 5.28%.

Over time, the prevalence of comorbidity components of the MetS had been increasing between the time periods 2014 and 2018 (Fig. 2). There were statistically significant differences in components of the MetS (hypertension, diabetes, obesity, and hyperlipidemia) between MetS and non-MetS groups (*P* = .000). The hyperlipidemia was the most determining component of MetS (odds ratio [OR] 14.83), whereas hypertension appeared to be the least (OR 6.31) (Table 2). Figure 3 showed the distribution of the numbers of positive components of MetS.

**Table 2:**
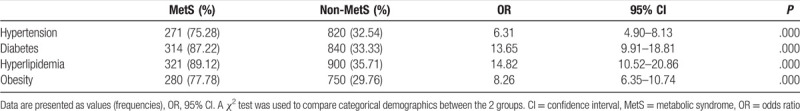
Prevalence of MetS components in the study population.

The presence of MetS was associated with higher rates for all studied comorbidities (Table 3). There were statistically significant differences in old cerebral infarction, chronic kidney disease, atrial fibrillation, and peripheral arterial disease between MetS and non-MetS groups (*P* < .05). The incidence of perioperative complications was significantly higher in the MetS group (121/360, 33.61%) than in the non-MetS group (427/2520, 16.94%, *P* < .001).

**Table 3:**
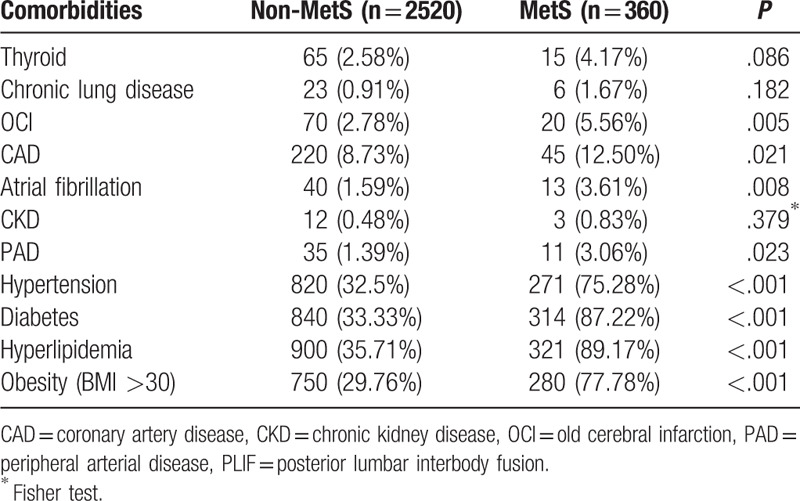
Prevalence of comorbidities in patients with and without MetS after PLIF.

Table 4 provided the results of postoperative complications. The patients with MetS was associated with an increased incidence of pulmonary complication (1.67% vs 0.63%, *P* = .035), pneumonia (1.94% vs 0.60%, *P* = .026), and cerebrovascular event (1.39% vs 0.44%, *P* = .023) compared with the patients without MetS (Table 4). The MetS group had a higher incidence of cardiac complications (*P* = .019), urinary tract infection (*P* = .018), postoperative ICU admission (*P* = .02), and DVT (*P* = .029) than non-MetS group. The superficial SSIs were observed in 6.94% of non-MetS patients and 11.11% of MetS patients (*P* = .03). The deep SSIs rates for the MetS and non-MetS groups were 2.22% and 0.79%, respectively, and this difference was statistically significant (*P* = .01). There were no differences in PE, MI, acute renal failure, reoperation, and death within 30 days between patients with MetS and individuals without MetS. Four (0.16%) patients in the non-MetS group and one (0.28%) patients in the MetS group died postoperatively. They all underwent a second operation, due to internal bleeding, respectively, and died in the ICU within 48 hours from second operation due to multiple organ failure. Patients without MetS required a mean hospital stay of 19.99 days compared to 22.16 days for patients with MetS (*P* < .001).

**Table 4:**
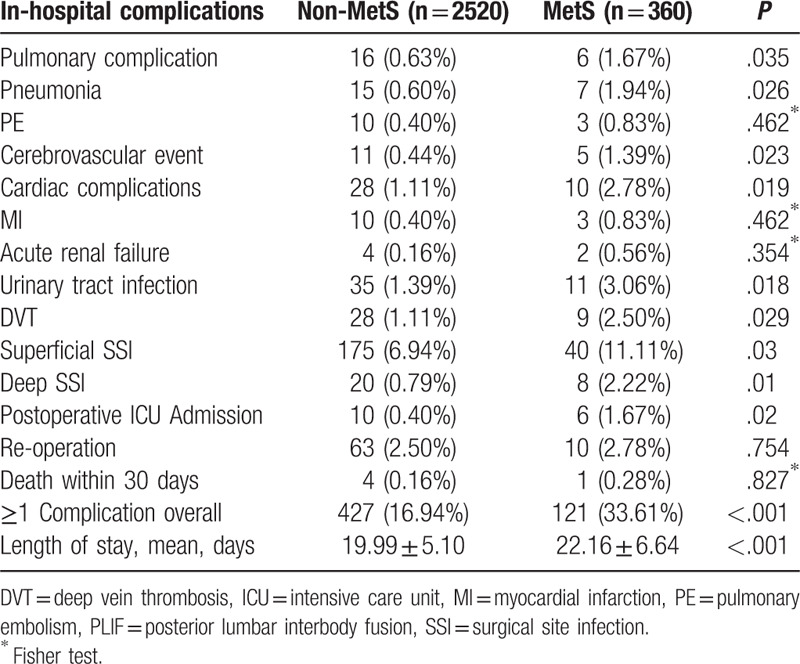
In-hospital complications in patients with and without MetS undergoing PLIF.

In the multivariable logistic regression models (Table 5), the MetS was a risk factor for the development of perioperative complications (OR: 1.31; 95% CI: 1.06–2.07, *P* < .001), and extended length of stay (ie, ≥75th percentile; OR: 1.69; 95% CI: 1.25–2.28, *P* = .001). The hypertension had increased length of stay (OR: 0.80, *P* = .009), and complications (OR: 0.71, *P* = .001). Meanwhile, we found that the dyslipidemia was a risk factor for the development of complications, and extend length of stay.

**Table 5:**
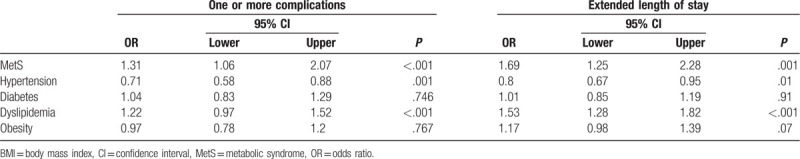
Adjusted multivariate logistic regression analysis for one or more complications and extended length of stay.

## 4 Discussion

In our study, the patients with MetS had a higher risk of perioperative complications, and prolonged length of stay after primary PLIF. Patients with MetS had an increased risk for cardiac events and pulmonary complications, urinary tract infection, cerebrovascular events, DVT, SSIs, and postoperative ICU admission compared with non-Mets group (*P* < .05). In the multivariable logistic regression, the MetS patients had a 1.31 higher odds (*P* < .001) and a 1.69 higher odds (*P* = .001) for postoperative complications, and extended length of stay.

Some studies assessed the impact of MetS on patients outcomes following spinal surgery. A 6696 patients study reported that the MetS was an independent risk factor for 30-day complications following the adult spinal deformity. The patients with MetS had higher rates of postoperative cardiac complications (*P* = 0.001), superficial surgical site infection (*P* = .004), sepsis (*P* = .009), re-operation (*P* = .006), pulmonary complications (*P* = .017), and prolonged hospitalization (*P* = .039).^[12]^ A study analyzed 1384 participants undergoing anterior cervical discectomy and fusion shown that the MetS was only significantly associated with an increased odds of a prolonged hospital stay ≥3 days (OR: 1.32; 95% CI: 1.12–1.56; *P* = .001); its presence did not significantly affect 30-day outcomes.^[13]^

After controlling the effect of obesity, Lovecchio et al^[14]^ shown MetS patients experienced higher rates of wound complications (3.8% vs 2.7%, *P* = .045), longer postoperative stays (29.1% vs 23.9%, *P* < .001), and higher readmission (7.4% vs 4.6%, *P* < .001) compared to obese controls following lumbar spine surgery. After controlling the total number of levels fused, Katherine et al^[15]^ reported that MetS patients increased postoperative complications (29.6% vs 12.8%; *P* = .001), including specifically neuro, pulmonary, urinary complications, and extended hospital length of stay (14.4% vs 6.4%; *P* < .001) after spine fusion surgery.

In an analysis of the National Inpatient Sample data from 2000 to 2008, Memtsoudis et al^[16]^ found MetS patients had significantly increased odds of major complications (OR: 1.11; 95% CI:1.03–1.20), longer hospital stay (OR:1.09; 95% CI:1.05–1.14), and high hospital costs (OR:1.25; 95% CI:1.19–1.31) undergoing lumbar spine fusion surgery.

The result was in agreement with previous studies which reported the prevalence of MetS to increase the risk of developing cardiovascular disease and pulmonary complications in patients.^[17,18]^ Glance et al found that perioperative mortality was doubled among super-obese patients with MetS after noncardiac surgery and cardiac adverse events were 2 to 2.5-fold higher in all patients with MetS undergoing no-ncardiac surgery.^[19]^

Past studies reported a link between pulmonary complications and MetS, can be partially explained by numerous proposed factors, such as low-grade systemic inflammation, sleep apnea, asthma, difficult airway, and obesity hypoventilation syndrome.^[20,21]^

The MetS was a significant risk factor for superficial SSIs (11.11% vs 6.94%, *P* = .03), and deep SSIs (2.22% vs 0.79%, *P* = .01). Indeed, it has been documented that MetS increases risk of superficial SSIs after liver resection by 70%^[22]^ as well as the risk of superficial, deep SSIs and wound dehiscence after infrainguinal bypass.^[23]^ The patients with MetS may cause an over-nutrition state providing a favorable environment, and were in a state of hormonal dysregulation and low-grade inflammation, likely contributing to a propensity for infections.^[24,25]^

The MetS patients had a higher rate of extended length of stay than control group. A plausible explanation for the finding could be that surgeons may be more cautious regarding postoperative care in these patients given the high-comorbidity burden posed by MetS.

Surprisingly, despite higher complication rates, there was no statistical difference in mortality between the MetS group and the non-MetS group (*P* = .827). It was possible that patients with MetS may be subject to more rigorous preoperative testing, thus leading to preselection of patients with MetS.

The mechanism of the MetS is not clear. Most of the clinical studies have reported the MetS stems from insulin resistance, which increased the risk of obesity and endothelial dysfunction.^[26]^ Obesity increases adipokines and hyperglycemia induces oxidative stress that lead to activation of the inflammatory and coagulation cascade. Specifically, endothelial dysfunction leads to the elevation of various cytokines, and CRP, predisposing individuals toward a proinflammatory, and prothrombotic state, and putting them at higher risks for perioperative complications.^[27,28]^

### 4.1 The limitation

There were several limitations in this study. The first limitation was the retrospective design that could have resulted in variability of data collection. Secondly, the definition of MetS was constantly evolving and had different diagnostic criteria. This definition was chosen to approximate published definition by the US National Cholesterol Education Program Adult Treatment Panel III,^[11]^ and was similar to the methodology reported by other investigators.^[2,29]^ The waist circumference was not routinely recorded in our data. So, the BMI was utilized over the standard waist circumference, which may underestimate the incidence of MetS. Thirdly, the incidence of MetS in our study may be underestimated. The information on patients with MetS treated with medication was not recorded. The participants with naturally adverse serum lipid and glucose profiles were classified as having normal serum measures, and potentially weaken the association between MetS and the PLIF. Finally, the diagnoses and procedures in the date were based on the *ICD-9-CM* coding system, and some of these codes may be redundant or interpreted differently by coders, thus clearly providing a source of bias.

## 5 Conclusion

In summary, this study provides evidence that patients with MetS increase risk for major perioperative complications, and extend length of stay following elective posterior lumbar spinal fusion. Further prospective studies are necessary to validate the results of our study as well as identifying specific postoperative outcome.

## Acknowledgments

The authors thank all the people who give the help for this study.

## Author contributions

**Conceptualization:** Xiaoqi He, Qiaoam Fei, Tianwei Sun.

**Data curation:** Tianwei Sun.

**Formal analysis:** Xiaoqi He, Qiaoam Fei.

**Funding acquisition:** Tianwei Sun.

**Investigation:** Xiaoqi He, Qiaoam Fei.

**Methodology:** Xiaoqi He, Qiaoam Fei.

**Project administration:** Tianwei Sun.

**Resources:** Tianwei Sun.

**Software:** Xiaoqi He, Qiaoam Fei.

**Supervision:** Tianwei Sun.

**Validation:** Xiaoqi He.

**Visualization:** Xiaoqi He.

**Writing – original draft:** Xiaoqi He, Qiaoam Fei.

**Writing – review & editing:** Xiaoqi He, Qiaoam Fei, Tianwei Sun.

## Abbreviations

Abbreviations: BMI = body mass index, DVT = deep vein thrombosis, ICU = intensive care unit, LDH = high-density lipoprotein cholesterol, MetS = metabolic syndrome, MI = myocardial infarction, NCEP-ATP III = National Cholesterol Education Program Adult Treatment Panel III, NIS = National Inpatient Sample, PE = pulmonary embolism, PLIF = posterior lumbar interbody fusion, SSI = surgical site infection, TG = triglycerides.

## References

[1] Sattar N McConnachie A Shaper A. Can metabolic syndrome usefully predict cardiovascular disease and diabetes? Outcome data from two prospective studies. Lancet 2008;371:1927 35. 18501419 10.1016/S0140-6736(08)60602-9

[2] Mraovic B Hipszer BR Epstein RH. Metabolic syndrome increases risk for pulmonary embolism after hip and knee arthroplasty. Croat Med J 2013;54:355 61. 23986276 10.3325/cmj.2013.54.355 PMC3760659

[3] Gandhi R Razak F Tso P. Metabolic syndrome and the incidence of symptomatic deep vein thrombosis following total knee arthroplasty. J Rheumatol 2009;36:2298 301. 19684153 10.3899/jrheum.090282

[4] Amin AK Clayton RA Patton JT. Total knee replacement in morbidly obese patients. Results of a prospective, matched study. J Bone Joint Surg Br 2006;88:1321 6. 17012421 10.1302/0301-620X.88B10.17697

[5] Wilson JA Clark JJ. Obesity: impediment to postsurgical wound healing. Adv Skin Wound Care 2004;17:426 35. 15492679 10.1097/00129334-200410000-00013

[6] Tzimas P Petrou A Laou E. Impact of metabolic syndrome in surgical patients: should we bother? Br J Anaesth 2015;115:194 202. 26109210 10.1093/bja/aev199

[7] Johnson LW Weinstock RS. The metabolic syndrome: concepts and controversy. Mayo Clin Proc 2006;81:1615 20. 17165640 10.4065/81.12.1615

[8] Chung AS Campbell D Waldrop R. Metabolic syndrome and 30-day outcomes in elective lumbar spinal fusion. Spine (Phila Pa 1976) 2018;43:661 6. 28858182 10.1097/BRS.0000000000002397

[9] Passias PG Brown AE Lebovic J. Metabolic syndrome has a negative impact on cost utility following spine surgery. World Neurosurg 2020;135:e500 4. 31857269 10.1016/j.wneu.2019.12.053

[10] Ford ES Giles WH Dietz WH. Prevalence of the metabolic syndrome among US adults: findings from the third National Health and Nutrition Examination Survey. JAMA 2002;287:356 9. 11790215 10.1001/jama.287.3.356

[11] Third Report of the National Cholesterol Education Program (NCEP) Expert Panel on Detection, Evaluation, and Treatment of High Blood Cholesterol in Adults (Adult Treatment Panel III) final report. Circulation 2002;106:3143 421. 12485966

[12] Ye IB Tang R Schwartz JT. Postoperative complications associated with metabolic syndrome following adult spinal deformity surgery. Clin Spine Surg 2020;33:E87 91. 31453837 10.1097/BSD.0000000000000859

[13] Teraguchi M Yoshimura N Hashizume H. Metabolic syndrome components are associated with intervertebral disc degeneration: The Wakayama Spine Study. PLoS One 2016;11:e0147565. 26840834 10.1371/journal.pone.0147565 PMC4739731

[14] Lovecchio F Fu MC Iyer S. Does obesity explain the effect of the metabolic syndrome on complications following elective lumbar fusion? A propensity score matched analysis. Global Spine J 2018;8:683 9. 30443477 10.1177/2192568218765149 PMC6232719

[15] Pierce KE Kapadia BH Bortz C. Operative fusion of patients with metabolic syndrome increases risk for perioperative complications. J Clin Neurosci 2020;72:142 5. 31899085 10.1016/j.jocn.2019.12.043

[16] Memtsoudis SG Kirksey M Ma Y. Metabolic syndrome and lumbar spine fusion surgery: epidemiology and perioperative outcomes. Spine (Phila Pa 1976) 2012;37:989 95. 22024892 10.1097/BRS.0b013e31823a3a13 PMC3288758

[17] Smolock CJ Anaya-Ayala JE Bismuth J. Impact of metabolic syndrome on the outcomes of superficial femoral artery interventions. J Vasc Surg 2012;55:985 93.e1. discussion 993. 22341577 10.1016/j.jvs.2011.10.109

[18] Ardeshiri M Faritus Z Ojaghi-Haghighi Z. Impact of metabolic syndrome on mortality and morbidity after coronary artery bypass grafting surgery. Res Cardiovasc Med 2014;3:e20270. 25478548 10.5812/cardiovascmed.20270 PMC4253799

[19] Watson K. Surgical risk in patients with metabolic syndrome: focus on lipids and hypertension. Curr Cardiol Rep 2006;8:433 8. 17059795 10.1007/s11886-006-0101-3

[20] Gautier A Bonnet F Dubois S. Associations between visceral adipose tissue, inflammation and sex steroid concentrations in men. Clin Endocrinol (Oxf) 2013;78:373 8. 22469460 10.1111/j.1365-2265.2012.04401.x

[21] Naik D Joshi A Paul TV. Chronic obstructive pulmonary disease and the metabolic syndrome: consequences of a dual threat. Indian J Endocrinol Metab 2014;18:608 16. 25285275 10.4103/2230-8210.139212 PMC4171881

[22] Bhayani NH Hyder O Frederick W. Effect of metabolic syndrome on perioperative outcomes after liver surgery: A National Surgical Quality Improvement Program (NSQIP) analysis. Surgery 2012;152:218 26. 22828143 10.1016/j.surg.2012.05.037 PMC3648869

[23] Arnaoutakis DJ Selvarajah S Mathioudakis N. Metabolic syndrome reduces the survival benefit of the obesity paradox after infrainguinal bypass. Ann Vasc Surg 2014;28:596 605. 24370499 10.1016/j.avsg.2013.10.015

[24] Tilg H Moschen AR. Adipocytokines: mediators linking adipose tissue, inflammation and immunity. Mat Rev Immunol 2006;61:772 83. 10.1038/nri1937 16998510

[25] Murphy AB Menendez ME Watson SL. Metabolic syndrome and shoulder arthroplasty: epidemiology and peri-operative outcomes. Int Orthop 2016;40:1927 33. 27138608 10.1007/s00264-016-3214-3

[26] Alessi MC Juhan-Vague I. Metabolic syndrome, haemostasis and thrombosis. Thromb Haemost 2008;99:995 1000. 18521499 10.1160/TH07-11-0682

[27] Ay C Tengler T Vormittag R. Venous thromboembolism—a manifestation of the metabolic syndrome. Haematologica 2007;92:374 80. 17339187 10.3324/haematol.10828

[28] Jamsen E Nevalainen P Eskelinen A. Obesity, diabetes, and preoperative hyperglycemia as predictors of periprosthetic joint infection: a single-center analysis of 7181 primary hip and knee replacements for osteoarthritis. J Bone Joint Surg Am 2012;94:e101. 22810408 10.2106/JBJS.J.01935

[29] Gandhi R Razak F Davey JR. Metabolic syndrome and the functional outcomes of hip and knee arthroplasty. J Rheumatol 2010;37:1917 22. 20634242 10.3899/jrheum.091242

